# Participation Profile of Children and Youth, Aged 6–14, with and without ADHD, and the Impact of Environmental Factors

**DOI:** 10.3390/ijerph18020537

**Published:** 2021-01-11

**Authors:** Tair Shabat, Haya Fogel-Grinvald, Dana Anaby, Anat Golos

**Affiliations:** 1 School of Occupational Therapy, Faculty of Medicine, Hebrew University, Jerusalem 91240, Israel; tair.shitiat@mail.huji.ac.il (T.S.); hayagrin@gmail.com (H.F.-G.); 2 School of Physical and Occupational Therapy, McGill University, Montreal, QC H3G 1Y5, Canada; dana.anaby@mcgill.ca

**Keywords:** children and youth, ADHD, participation, frequency, involvement, environment, well-being

## Abstract

Background: Children and youth with attention deficit hyperactivity disorder (ADHD) may experience difficulties in participation, but few studies examine their participation and the environmental factors affecting participation. This study explored the participation and the environmental factors of children and youth, with and without attention deficit hyperactivity disorder (ADHD), in the following three settings: home, school, and community. Materials and Methods: Parents of 65 participants aged 6–14 (M = 9.91, SD = 1.87) with and without ADHD completed the Participation and Environment Measure for Children and Youth (PEM-CY) questionnaire, which evaluates participation and environmental factors, along with demographic and screening questionnaires. Results: The ADHD group (*n* = 31) scored significantly lower than the non-ADHD group (*n* = 34) in “frequency” at home, “involvement”, and overall environmental support in all settings, with parents expressing a greater desire to change their child’s home and community participation. For the ADHD group, a relationship was found between environmental support and involvement in all three settings. Conclusions: The findings demonstrated differences in the participation of children and youth with ADHD across different settings, compared to those without ADHD, and confirmed the effect of environmental factors on participation, especially involvement. It is essential to consider participation measures and environmental factors when designing interventions for children and youth with ADHD.

## 1. Introduction

The International Classification of Functioning, Disability and Health (ICF) of the World Health Organization (WHO) defines participation as “involvement in a life situation” [1], which is considered an important outcome measure for rehabilitation and intervention [1,2], as well as the focus and goal of many health and rehabilitation disciplines [3]. Participation in meaningful activities has a positive influence on health and well-being and is essential for the development of a person’s abilities and self-efficacy [4,5], as well as for skill acquisition and learning among children and youth [6]. Participation is a multidimensional concept that includes various dimensions such as frequency and involvement. Participation frequency is considered as an objective aspect, referring, for example, to the number of times a person participates in an activity [7], while involvement refers to the feelings and personal experience of participation and includes various elements such as motivation, adherence, satisfaction, and emotional engagement [7,8,9,10].

The participation of children and youth with different health conditions (such as attention deficit hyperactivity disorder (ADHD) and autism spectrum disorder (ASD) and/or disabilities was found to be limited, compared to that of children with typical development [11,12,13]. For example, it was found that children with disabilities participate less frequently and/or are less involved in activities in the home, school, and community settings, compared to their peers with typical development [14,15,16].

One of the health conditions that may affect the participation of children and youth in different settings is attention deficit hyperactivity disorder (ADHD). This is a neurodevelopmental disorder characterized by attention deficit and/or impulsivity and hyperactivity, whose prevalence among children and youth is estimated at about 5%. Symptoms of ADHD persist in adulthood, with prevalence among adults estimated at about 2.5% [17,18]. A diagnosis of ADHD is based on the appearance of six or more criteria (such as lack of attention to details, difficulty organizing tasks, etc.), related to inattention, hyperactivity, and/or impulsivity, some of which appear prior to age 12; and these symptoms must occur in at least two life environments in a way that impairs functioning and quality of life [17].

ADHD has far-reaching and long-term consequences in all functioning areas, as it affects various aspects of a person’s life, including daily functioning, employment, social participation, and family stability [17,18]. Studies among ADHD populations have often focused on specific impairments and/or functioning areas in which the implications of ADHD arise. For example, Shimoni et al. [19] and Engel-Yeger and Ziv-On [20] reported that children with ADHD participate less frequently in most leisure activities, receive less enjoyment from formal leisure activities, and show a lower preference for participation in some leisure activities, such as physical and social leisure activities, compared to children without ADHD. Additional studies indicated difficulties in social functioning of children and youth with ADHD compared to their peers [21,22], affecting their participation in various social activities [19,20].

Social difficulties of children and youth with ADHD may include peer rejection, inappropriate behavior, and/or difficulties with social skills such as collaboration, taking turns, reciprocity, and focus on conversation and play [21,22]. In addition, this population may experience difficulties in academic functioning [23,24], putting them at greater risk for low academic achievement, suspensions and expulsion from school, more absences, and even dropping out permanently, compared to populations without ADHD [25,26]. While these studies focus on one area of functioning, Harpin [27] described difficulties in the participation of a population with ADHD in multiple settings, and Lavi et al. [28] also reported significantly lower participation of adolescents with ADHD in daily activities and in school and home participation, compared to their peers without ADHD.

In summary, most studies examining the functioning of children and youth with ADHD are often focused on their specific difficulties and disabilities rather than their overall participation. In addition, there are few studies describing the participation profile of children and youth with ADHD compared to their peers without ADHD, particularly with respect to different settings (home, school, and community). Thus, a need exists to expand professional knowledge of the effects of ADHD on the participation of children and youth with this diagnosis, as it impacts their daily life in various settings [19].

In addition to health conditions, various environmental factors, such as physical and sociocultural factors, may also affect a person’s development and participation [1,3,29,30]. Environmental factors can either support or hinder (erect barriers to) participation [3]. It is therefore important to examine the environmental factors and their impact on participation [31]. Not surprisingly, people with disabilities often identify relationships between environmental factors, participation, and quality of life. This highlights the need to assess the environmental impact on their participation at the community and social levels [29]. Research has found that the environmental domains noted in the ICF influence the participation of children with disabilities [32]. For example, the study by Bedell et al. [14], which examined community participation of children with and without disabilities, indicated that parents of children with disabilities more often rated environmental factors as barriers to participation and more rarely as supports, compared to parents of children without disabilities. Furthermore, it appears that the environment plays a unique role in influencing participation in different settings (home, school, and community). Specifically, environmental barriers were found to directly affect the frequency of participation and involvement in all settings, whereas environmental supports only influenced involvement in home and community settings, and participation frequency in the community setting [11].

However, despite increasing research on the contribution of environmental factors in explaining the participation of children and youth with disabilities, the majority of these studies have focused on children with physical disabilities [32], autism [33], or developmental coordination disorder (DCD) [34]. A number of studies conducted among children and youth with ADHD have addressed the relationship between environment and functioning. They have identified factors such as attitudes and social–family environment as relevant in conducting a functional assessment of this population [35,36]. One qualitative study showed that over half of the participants described a particular aspect of ADHD as context-dependent, which may indicate an association between environment and ADHD symptoms [37]. In a review article, Dvorsky and Langberg [38] reported that social and family support, particularly social acceptance and positive parenting, has a positive effect and can even prevent the negative effects of ADHD. They also noted that research examining such supportive and protective factors is still in its infancy, suggesting further exploration.

All this points to the need for an in-depth examination of how environmental factors impact the participation of children and youth with disabilities [32]. Specifically needed is a comparison of those with ADHD to their peers without ADHD, in a range of settings, with attention given to the relationship between environmental supports and participation patterns. Our study addressed this need by examining the participation profile of children and youth with ADHD, and the environmental factors that may influence their participation. The results may contribute to a deeper understanding of the participation of children and youth with ADHD, thereby assisting in assessment and contributing to the development and implementation of appropriate intervention programs for this population.

The present study focuses on examining the participation of children and youth (aged 6–14) with and without ADHD, in home, school, and community settings, identifying the environmental supports for and barriers against participation, and examining the availability of supporting resources. Our specific objectives were to examine the following: (a) the differences between children and youth with and without ADHD with respect to participation patterns (in terms of frequency, involvement), desire for change, and the overall environmental support in each of the settings (home, school, and community); (b) the relationship between the overall environmental support and the participation patterns (frequency and involvement) in the different settings in each group (with and without ADHD); and (c) the differences in the participation patterns between the different settings (home, school, and community), and to describe the environmental factors (support and barriers) that influence participation of children and youth with ADHD.

## 2. Materials and Methods

### 2.1. Study Design

A descriptive quantitative and comparison cross-sectional design was used.

### 2.2. Participants

The study population included 65 parents of children and youth, aged 6–14, most of them (about 60%) from urban areas in the central district of Israel, who were recruited by voluntary response sampling. Most parents were married (89.5%), were aged 45 and under (mothers: 73.4%; fathers: 65.1%), and had an academic education (mothers: 83.6%; fathers: 74.6%). The participants were divided into the following two groups: (a) an ADHD group (31 participants) and (b) a non-ADHD group (34 participants) matched in age range and adjusted for gender and socioeconomic status (according to the level of family income). The inclusion criteria for each group were children and youth whose parents reported that they did (ADHD group) or did not (non-ADHD group) receive a diagnosis of ADHD from a qualified professional, with all reports confirmed by the ADHD Questionnaire (see Instruments, Section 2.3). The exclusion criteria for both groups were as follows: (a) the parents’ lack of fluency in the Hebrew language; (b) attendance of the child/adolescent in a special education environment; and (c) one or more of the following diagnoses for the child/adolescent: cerebral palsy, autism, epilepsy, Tourette syndrome, intellectual disability, psychiatric disorder, and/or brain injury, according to their parents’ report in the demographic questionnaire (see Instruments, Section 2.3). In the ADHD group, at least half of the parents reported learning, sensory, and emotional–behavioral difficulties of their children. As seen in Table 1, both groups included participants with an average age of 9–10 years, most were boys (over 64%), and were from average or high socioeconomic strata. No significant differences were found in the characteristics between the two groups with respect to age, gender, or family socioeconomic status.

### 2.3. Instruments

#### 2.3.1. A demographic Questionnaire

A demographic questionnaire was developed for this study as a parental reporting tool. Its purpose was to characterize the study population, as well as to provide information about the child/adolescent and his/her family. The personal details in the questionnaire included items such as age, gender, country of birth, educational framework, health condition, and family income.

#### 2.3.2. The Attention Deficit and Hyperactivity Screening Questionnaire

The Attention Deficit and Hyperactivity Screening Questionnaire [17] is a parent-report questionnaire that identifies symptoms of ADHD according to the criteria found in the Diagnostic and Statistical Manual of Mental Disorders, Fifth Version (DSM-5) [17]. It includes 18 criteria rated by the parent on a scale of 4 grades (3 = “very much”, 0 = “not at all”). The questions are divided into criteria related to attention and hyperactivity, with suspected ADHD indicated by a score of 2 or higher in at least 6 of the 9 criteria for attention, and/or in at least 6 of 9 criteria related to hyperactivity and/or impulsivity. This questionnaire was used as an exclusion criterion for the non-ADHD group.

#### 2.3.3. The Participation and Environment Measure for Children and Youth (PEM-CY)

The Participation and Environment Measure for Children and Youth (PEM-CY) [8] is a parent-report instrument that examines participation and environmental factors affecting the participation of school-age children (5–17 years of age) across the following three settings: home, school, and community. The PEM-CY participation items represent broad types of activities typically performed in each setting, i.e., home (10 activities), school (5 activities), and community (10 activities). For each activity type, parents are asked to note the following: (a) how frequently their child participates (“never” = 0 to “daily” = 7); (b) how involved their child is while participating (“minimally” = 1 to “very” = 5); and (c) whether they desire change in their child’s participation (“no” or “yes”; if “yes”, parents identify the type of change desired: “frequency”, “involvement”, and/or “variety”). Parents are then asked whether certain features of the environment help or hinder their child’s participation in activities in each setting (“not an issue”, “usually helps”, “sometimes helps/makes harder”, “usually makes harder”). They are also asked about perceived adequacy/availability of supporting resources (“not needed”, “usually yes”, “sometimes yes/no”, “usually no”). The PEM-CY has been found to have moderate-to-good internal consistency (Cronbach’s α = 0.59–0.83) in the participation scales. Its test–retest reliability was found to be moderate at school (r = 0.58), and high at home (r = 0.84) and in the community (r = 0.79). Additionally, high reliability was found in the environment scale (r > 0.76). This measure identifies significant differences in participation patterns and environmental factors between children with and without disabilities [12], supporting its construct validity. It also has been effectively used in the Israeli context.

### 2.4. Procedure

This study was approved by the Ethics Committee of the Hebrew University, Jerusalem, Israel (No. 27032018). Ads for recruiting subjects were posted on social networks and relevant forums; for the ADHD group, therapists working with children and youth with ADHD were also contacted. Parents who showed interest and agreed to participate in the study received an explanatory letter and filled out the questionnaires electronically, via email, or manually, according to their preference. The data were collected without identifying personal and/or computer information. Screening of the returned questionnaires was performed according to the exclusion and inclusion criteria (see Participants, Section 2.2).

### 2.5. Data Analysis

Statistical analysis was performed using the SPSS version 25.0 (Statistical Package for the Social Sciences, Armonk, NY, USA) [39], with a significance level of 0.05. Descriptive statistics were used to describe the study population, including participants’ background data distribution, and the environmental factors (supports and barriers) impacting the ADHD group. Differences between the two groups in gender and socioeconomic status were examined using the chi-squared test for independence, and an independent-samples *t*-test was used for the age variable. For each setting (home, school, community), the participation patterns (frequency and involvement) were measured using mean variables. The desire for change in their child’s participation was measured as the percent of activities in which the parents indicated that desire. In addition, according to the PEM-CY manual’s guidelines, a variable was calculated for the overall environmental factors supporting participation (“overall environmental support” (in each setting separately. All environment questions were recoded into 3-point scale by merging “Not an issue/Not needed” with “Usually helps/Usually yes”. The sum of all the environment ratings was divided by the maximum possible score within a setting, and multiplied by 100 (higher scores indicated more support of children’s participation or more availability of the supporting resource). In order to compare the participation patterns, the desire for change, and the overall environmental support between the two groups (with and without ADHD), one-way MANOVA analysis was used. Effect-size calculation was conducted using partial eta squared, with η^2^ > 0.14 defined as high effect, 0.06 < η^2^ < 0.13 as medium, and 0.01 < η^2^ < 0.05 as low [40]. In order to examine the relationship between the overall environmental support and the participation patterns (frequency and involvement) within each group, Pearson’s correlations were calculated. In order to examine the differences among the three settings in the participation patterns (frequency and involvement) of the ADHD group, a one-way repeated measures ANOVA was used with Bonferroni correction.

## 3. Results

### 3.1. Comparison of Participation and the Overall Environmental Support between Groups in the Different Settings

Differences in participation patterns between the two groups were examined using a one-way MANOVA analysis. The differences between the groups on the combined dependent variables were statistically significant for frequency (*F*_(3, 61)_ = 3.91, *p* = 0.013; partial η^2^ = 0.16) and for involvement (*F*_(3, 61)_ = 14.16, *p* < 0.001; partial η^2^ = 0.41). As seen in Table 2, follow-up univariate ANOVAs showed significant differences with medium-to-high effect sizes in the frequency aspect in the home setting, and in the involvement aspect in all three settings. That is, the ADHD group was found to participate less frequently at home and was less involved in the three settings, compared to the non-ADHD group. It should be noted that no significant differences were found between the groups in the frequency aspect in the school setting.

The combined dependent variable of the desire for change significantly differs between the two groups (*F*_(3, 61)_ = 4.83, *p* = 0.004, partial η^2^ = 0.19). In follow-up univariate ANOVAs, significant differences were found between the groups with medium-to-high effect sizes in the home and community settings, meaning that parents in the ADHD group were more interested in change than parents in the non-ADHD group at home and in the community, but not at school.

Examining the differences between the groups with respect to the overall environmental support, a significant difference between groups was found for the three combined settings (F _(3, 60)_ = 13.39, *p* < 0.001, partial η^2^ = 0.40), along with significant differences with high effect sizes that were found between the two groups in each setting. Thus, the ADHD group reported the overall environmental support to be lower than the non-ADHD group, indicating that, among the ADHD group, the environmental factors are less supportive of children’s participation or are less available to lend support.

### 3.2. Correlation between Overall Environmental Support and Participation Patterns in all Settings and for Each of the Groups

**Figure 1:**
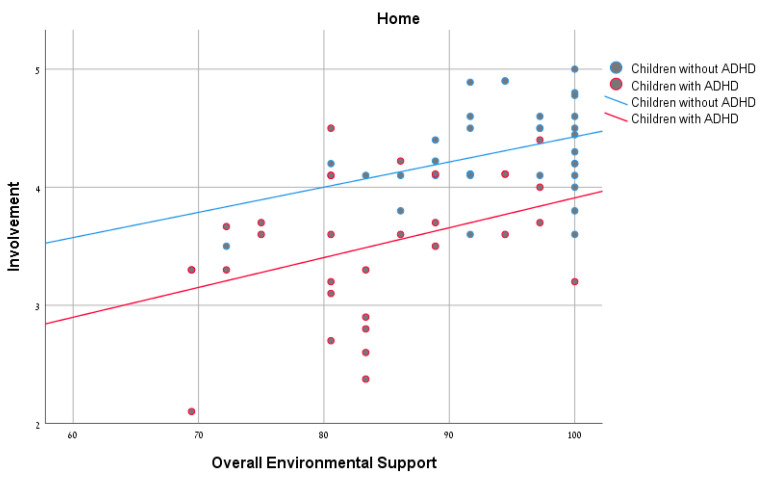
Correlations between overall environmental support and involvement among children with and without attention deficit hyperactivity disorder (ADHD) in the home environment.

**Figure 2:**
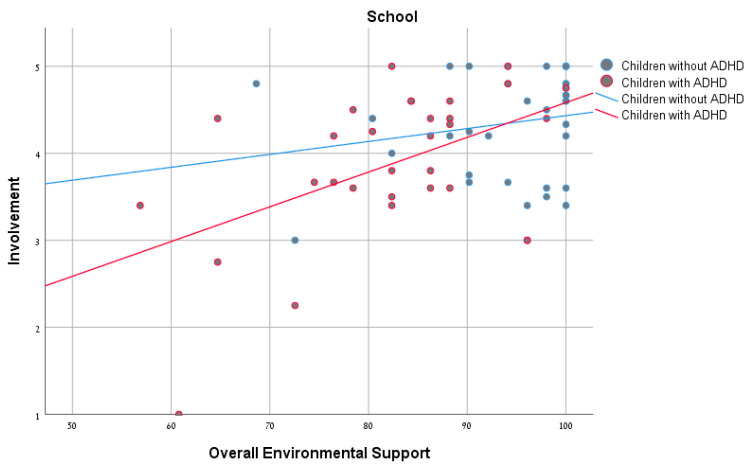
Correlations between overall environmental support and involvement among children with and without ADHD in the school environment.

**Figure 3:**
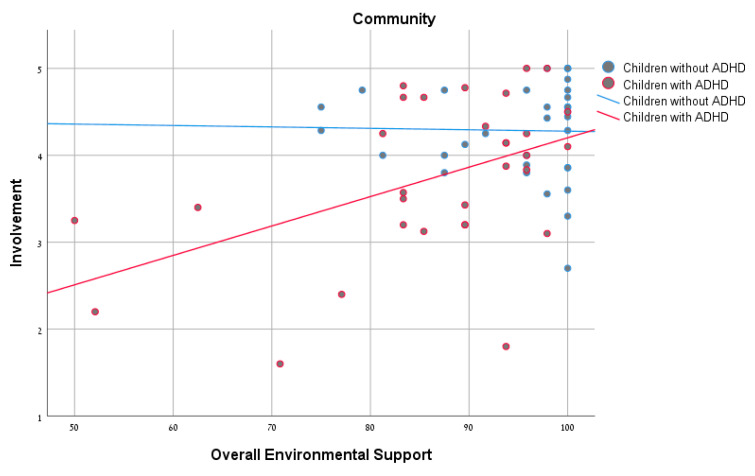
Correlations between overall environmental support and involvement among children with and without ADHD in the community environment.

Pearson’s correlations were calculated to explore the relationships between environmental support and the participation patterns for each of the groups. As seen in Table 3, no significant correlation was found between the overall environmental support and frequency of participation. However, significant positive correlations were found between overall environmental support and involvement of both groups in the home settings (Figure 1). In the school and community settings, significant positive correlations were found for the ADHD group, but not for the non-ADHD group (Figure 2 and Figure 3). In conclusion, the ADHD group showed a positive and significant relationship between overall environmental support and involvement in each of the settings, whereas with the non-ADHD group a similar relationship was found only in the home setting.

### 3.3. Differences in Participation Patterns and Prevalence of Environmental Factors Impacting the ADHD Group

**Figure 4:**
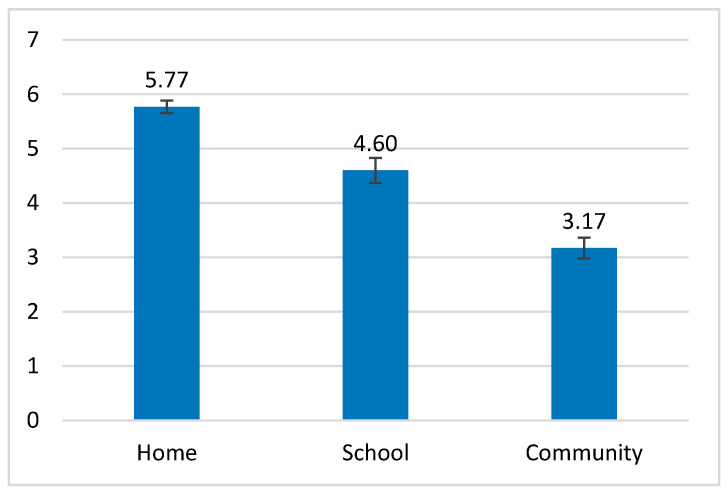
Frequency of participation of the ADHD group in the three settings.

One-way repeated measures ANOVAs were conducted to determine whether there was a statistically significant difference in the ADHD group’s participation among the different settings. Frequency of participation significantly changed with the settings as follows: *F*_(2, 60)_ = 79.738, *p* < 0.001, partial η^2^ = 0.73. Pairwise comparisons with Bonferroni correction showed all three settings significantly differed from one another (*p* < 0.001). The frequency of participation at home was found to be higher than in the school and the community environments, and the frequency of participation in the school was found to be higher than in the community (Figure 4).

**Figure 5:**
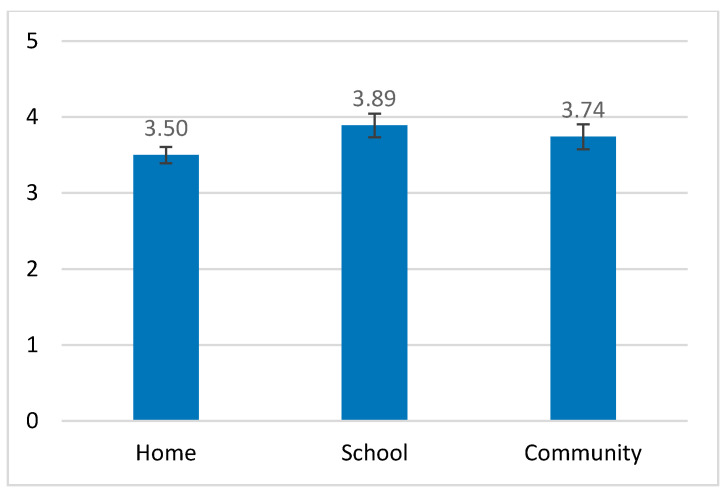
Involvement in participation of the ADHD group in the three settings.

Involvement in participation also significantly changed with the settings as follows: *F*_(2, 60)_ = 3.943, *p* = 0.025, partial η^2^ = 0.12. In pairwise comparisons with Bonferroni correction, the mean of involvement at home was significantly lower than in school (*p* < 0.05), but no significant differences were found when comparing the involvement at home and school to the involvement in the community (Figure 5).

The ADHD group’s description of supports and barriers affecting participation in the different settings was evaluated by calculating the percentage of participants’ consensus. Examining the supports, it appears that, in the home environment, “the attitudes and actions of therapists and other professionals” were reported as the most supportive factor (27.60%). In the school environment, the most supportive factor reported was “relationships with peers” (32.30%), followed by “the attitudes and actions of teachers, coaches, or staff” (25.80%). The result was similar for the community environment, where the most supportive factor for the ADHD group was “attitudes and actions of other members of the community” (29%).

Examining the barriers of the ADHD group, it was found that, in the home environment, “the cognitive demands” were reported as the most common inhibitors (38.70%). In the school environment, “the cognitive demands” and “the sensory stimulation” were the most frequently reported inhibitory factors (45.20% for each of them). Similar inhibitory factors were reported in the community environment, leading with sensory stimulation (19.40%) and cognitive demands (16.10%).

## 4. Discussion

This study was designed to examine the participation profile as affected by environmental factors (supports and barriers) among children and youth with ADHD, compared to their peers without ADHD. Additionally, the study set out to examine the relationship between overall environmental support and participation patterns (frequency and involvement) in each group, in the following three settings: home, school, and community.

### 4.1. Comparison of Participation Patterns, Desire for Change, and Overall Environmental Supports

Regarding the comparison of the participation patterns and the desire for change, it was found that, in the home environment, children and youth with ADHD participated less frequently and were less involved than their peers without ADHD. These findings are consistent with previous research suggesting that children with disabilities participate less frequently and are less involved at home, compared with typically developing children [16]. They also support the findings of Lavi et al. [28], showing that adolescents with ADHD participate less in the home environment than their peers without ADHD. In addition, as expected, our study found that parents of children and youth with ADHD reported a greater desire for change in their children’s participation in the home environment, compared to the non-ADHD group. This suggests that the parents of children with ADHD were less satisfied with their child’s participation patterns. A possible explanation for this is the difficulty in balancing the stability of the family with the need to assist a child/adolescent with ADHD [27,41], which is made even more challenging by parents’ exposure to their child’s ever-present difficulties in this environment.

Regarding the school setting, our findings indicated that children and youth with ADHD were less involved than children and youth without this diagnosis. This may be due to the difficulties in the social functioning of children and youth with ADHD, which comprise four of the five school-related activities in the PEM-CY questionnaire. Indeed, other studies of children with ADHD indicate high rates of peer rejection, low teacher ratings of their social skills in the classroom, difficulty in social involvement, along with difficulty in cooperation and in reciprocal conversation while playing with others [21,22]. Additionally, this finding was consistent with a previous study indicating that children and youth with disabilities (including ADHD) are less involved in the school environment than their typically developing peers [15].

However, in our study, no significant difference was found between the groups in frequency of participation in the school environment. A possible explanation for this is that participation frequency is influenced by school policy and routine [15], which may possibly obscure the differences between the groups. In addition, no significant differences were found between the groups in the desire for change in school environment participation. This may be related to the fact that the school environment is less accessible or familiar to parents.

Regarding the community environment, our findings indicated that the ADHD group was less involved than the non-ADHD group. This is similar to the study by Engel-Yeger and Ziv-On [20], which found that children with ADHD preferred to participate less in most leisure activities, and also received less enjoyment from formal leisure activities, compared to children without ADHD. Since involvement typically includes elements such as motivation and personal preference, which can be considered participation-related constructs [10], it may be assumed that the lower preference among children with ADHD to participate in the leisure activities that most often occur in the community environment would affect their involvement here. However, our study found no significant difference in frequency of participation between groups in the community environment. This finding is not consistent with the study of Bedell et al. [14], according to which children and youth with disabilities participate less frequently and are less involved in community activities than typically developing peers. A possible explanation is the difference in group characteristics, that is, the study described above included diagnoses in addition to ADHD, such as orthopedic defects, developmental delay, and autism, which may affect both the frequency and the ability to participate in activities. In addition, our study showed that parents of children and youth with ADHD reported a greater desire for change in their children’s participation in the community, compared to those without ADHD. A possible explanation is that parents perceive themselves as influencing their children’s participation in community activities, given their role in registering their children for such activities and encouraging them to participate.

Overall, our findings indicated lower involvement in the ADHD group compared to the non-ADHD group in all three settings. Since the construct of involvement refers to the level of concentration, emotional involvement, satisfaction, and attention when performing an activity [8], it is not unlikely that children and youth with ADHD will experience difficulty in maintaining attention and active partnership throughout a particular activity. In accordance with the lower involvement of children and youth with ADHD in all settings, the implementation of training programs for parents and teachers, as well as outreach programs in the community setting, can be beneficial for promoting their participation, personal involvement, and well-being. Regarding overall environmental support, this study found a significant difference between the groups in each of the three settings, meaning that environmental factors were less supportive of, or were less available to lend support to, the participation of children and youth with ADHD, compared to the non-ADHD group. These findings support the literature, which documents differences in the environmental support given to children with and without disabilities in different settings, and highlights the fact that there are more environmental barriers that affect the participation of children with disabilities, compared to children without disabilities [12,13]. A specific example of this is reflected in the study by Coster et al. [15], who found that parents of students with disabilities were significantly more likely to report patterns of the school environment that hindered participation, and that the resources needed to support their child’s participation were not adequate, compared to students without disabilities.

### 4.2. Relationship between Overall Environmental Support and Participation Patterns

The results of the study found no significant association between environmental supports and participation frequency, for either of the groups (with and without ADHD). A similar finding was reported in the study of Rosenberg et al. [30], which examined the effect of environmental barriers on participation among children with mild developmental disabilities and found no significant correlation between environmental barriers and the number of activities in which the child participated (diversity) or the child’s participation frequency (intensity). These results may be explained by the fact that the ADHD group can easily attend an activity, since they do not necessarily need major accessibility. However, our study did find a significant association between environmental support and involvement in all three settings among children and youth with ADHD, and in the home setting among children and youth without ADHD. It seems that, for the ADHD group, being involved (which means to be fully immersed in the activity) can be more challenging. This reinforces the understanding that participation is a multidimensional concept whose assessment requires addressing various aspects [7,9,31], including frequency and subjective measures such as involvement.

Our findings demonstrated a positive connection between environmental support and involvement among children and youth with ADHD in all the settings, confirming the need for environmental support to promote participation. In light of the importance of participation and its contribution to development, health, and well-being of children and youth, further examination is advisable to better understand the effects of increased participation on the well-being of this population.

It should be noted that, in the ADHD group, a significant correlation was found in all three settings, whereas in the non-ADHD group the correlation was found to be significant only in the home environment. These differences between groups indicate that, for children and youth with ADHD, the environmental supports are more significant influences on their involvement in activities in different settings.

### 4.3. Comparison of Frequency and Involvement between the Different Settings and a Description of the Environmental Factors

In comparing the different settings for frequency and involvement patterns, the home was found to be the environment in which children and youth with ADHD participated most frequently, yet they were less involved. A possible explanation for this is that the home environment is the main place where most daily tasks are performed [42]. As such, it contains activities that may take place in the family routine at a high frequency, such as household chores (e.g., setting the table and cleaning the room) and personal care management (e.g., maintaining hygiene and brushing teeth), as well as other unstructured informal activities, such as play, arts and crafts, and getting together with other people, that often require a child’s initiative. At the same time, the child/adolescent may be dependent on another person, particularly his or her parents, when performing activities in this environment [34], which may result in lower involvement. An example of this was given in the study of Dunn et al. [43], who examined participation among children and youth with ADHD in various household tasks, and highlighted their need for support from family members while performing them; this tendency may also affect other activities reflected in the present study.

In contrast, school was found to be an environment where children and youth with ADHD are mostly involved in school activities, which may be related to the school activities themselves that are more structured. Our results, however, were limited to activities included in the PEM-CY questionnaire, which do not necessarily highlight the difficulties of this population in executive functions [44,45] and academic functioning [24,26]. These difficulties are underrepresented in the questionnaire items related to school activities, but are more prominent in home activities such as homework preparation, school preparation, and household chores.

According to our results, the community showed the lowest frequency of participation among children and youth with ADHD. This may result from the nature of community activities, such as group events or traveling, which may occur less frequently than home and school activities. Significantly, this low frequency was also reported by the non-ADHD group, an outcome supported by various studies that used the PEM-CY and similarly indicated highest participation frequency at home and lowest in the community [12,13,34]. However, it is important to note that these studies did not examine the frequency differences among the three settings, but rather compared groups with and without disabilities in different participation patterns.

Examining the environmental supports and barriers influencing children and youth with ADHD indicated that the factors which stood out most frequently as inhibitors, in all three settings, were the activity demands, and in particular the cognitive demands (e.g., concentration, attention, and problem-solving). This finding is consistent with the cognitive difficulties of the ADHD population related to attention, concentration [17], and executive functions, all of which impair their participation in various occupations throughout the day [28]. In addition to these, social demands (at home and school) and physical demands (in the community) were also reported as inhibiting participation among this population. The findings relate to activity demands, given the confirmed interactions between the person, the environment, and the activity [14]; therefore, changing the activity demands in the environment may promote participation. Interventions that include this adaptation of activity demands may promote the participation of children and youth with ADHD in the various settings. Adaptations in the activity demands, and in particular in the cognitive demands, can be applied by professional training and guidelines to parents, teachers, and community members who are involved in the participation of this population.

Another major barrier for children and youth with ADHD in school and the community is the sensory stimulation (e.g., amount and/or type of sound, noise, light, temperature, textures of objects, and crowds). This refers to distractions due to unrelated stimuli, and it may therefore reflect the high prevalence of comorbidity associated with sensory modulation dysfunction [46]. Reports of sensory interference highlight the need to assess environmental sensory conditions (for example, by using the Sensory Processing Measure and/or the Sensory Profile Questionnaire) and to consider them when designing interventions, in order to promote participation. Reducing sensory stimuli such as classroom decorations, and/or performing community activities in a relatively quiet environment, may be examples of such sensory adjustments.

Regarding the supports, other people’s attitudes and actions were found to promote the participation of children and youth with ADHD in the three settings (e.g., babysitters and other professionals at home; teachers and staff at school; and members of the community, such as shopkeepers and instructors). Indeed, according to the literature, positive attitudes in the community and culture can promote participation [32], with the strongest evidence for social protective factors being found in social acceptance having a positive effect on the symptoms of the disorder [38]. In addition, relationships with peers emerged as one of the strongest supports for children and youth with ADHD. The fact that this factor was found to be helpful in school was interesting, given that it is adult support through their presence and supervision that is sometimes perceived as providing confidence among children with ADHD in the school environment [47].

Given that the environment is a potentially modifiable factor, there is considerable value in identifying which features of the home, school, and community environments are barriers to participation, so that interventions can be directed appropriately [7]. Therefore, these findings that indicate specific environmental factors constituting supports (people’s attitudes and actions, and relationships with peers) or barriers (activity demands and sensory stimulation) to participation of children and youth with ADHD may greatly contribute to the well-being of that population.

### 4.4. Research Limitations and Recommendations for Further Research

The present study has a number of limitations, for which further research is recommended. First, the study focused on a convenience sample of 65 children and youth with and without ADHD aged 6–14 years, which is a wide age range. Moreover, most of them have average or above-average socioeconomic status, live in the central district of Israel, and have parents with an academic education. Further studies need to include a larger and more representative sample of the two groups, including smaller age ranges, various socioeconomic strata and areas of residence, with varying levels of parents’ education, in order to enable better generalization of the findings. In addition, further studies should include children and youth with various health conditions, following the literature related to the range of health-related characteristics among representative study samples. Second, the information was based on parents’ reports regarding their children’s diagnosis of ADHD and the study criteria. Further research could include additional information from a professional regarding the ADHD type and comorbidity, such as sensory modulation dysfunction, learning disabilities, and/or DCD, which is common in this population [27,46,48]. Additionally, it is recommended to include other perspectives, such as the child/youth themselves and/or others (teachers and caregivers), especially regarding settings outside the home. It should also be noted that parents with ADHD are more likely to have children with ADHD [27]; this may affect parents while answering a long questionnaire like the PEM-CY, as well as their responsiveness to participating in the study. Therefore, it is recommended to substitute or add tools, including semi-structured interviews with parents, in order to facilitate questionnaire completion and deepen their understanding of the participation patterns. Moreover, this study included one measure for evaluating participation, which is a multidimensional and complex concept that no single dimension of measurement is likely to fully capture [7]. In following up the study results, further research should use additional measures related to the more subjective patterns inherent in this concept. Finally, further examination of the environmental supports and barriers, as well as the impact of environmental supports on the involvement and well-being of children and youth with ADHD, may also contribute to the expansion of professional knowledge.

## 5. Conclusions

This study described the participation profile, environmental factors (supports and barriers), and the relationship between overall environmental support and the participation patterns of frequency and involvement, among children and youth with and without ADHD, aged 6–14, based on parental reports. The findings indicated that, compared to their peers without ADHD, children and youth with ADHD participate less frequently in the home setting, they are less involved in all three settings, and their parents are more interested in changing their participation at home and in the community. Information about the specific activities to which parents want to see change is clinically important, as it can guide goal setting and targeted intervention. At the same time, children and youth with ADHD reported lower overall environmental support. Our findings showed a relationship between environmental support and involvement of children and youth with ADHD in all three settings, in contrast to children and youth without ADHD. These differences between groups reflect the interactional nature of participation, while confirming the need for environmental support to promote participation mainly among children and youth with ADHD.

In addition, differences in their participation patterns were found in various settings. These findings highlight the need for a broad examination of participation in different settings. As mentioned above, since participation is a multidimensional concept, its assessment requires addressing multiple aspects, including subjective measures such as involvement.

Different environmental factors were found to support or inhibit the participation of children and youth with ADHD, such as other people’s attitudes and actions, relationships with peers, activity requirements (particularly cognitive), and sensory stimuli. This knowledge can lead to a greater effort to evaluate environmental support for children and youth with ADHD and improve their participation patterns (particularly their involvement) in various activities in different settings. The resulting development of intervention programs will benefit this population and contribute to their well-being.

## Figures and Tables

**Table 1:** Demographic characteristics of the participants.

		Children and Youth with ADHD		Children and Youth without ADHD			
		*n*	Mean (SD)	*n*	Mean (SD)		
Age		31	10.37 (1.83)	34	9.48 (1.83)	t_(63)_ = −1.95 ^a^	*p* = 0.056
		***n***	**%**	***n***	**%**		
Gender	male	20	64.52	22	64.71	χ^2^_(1)_ = 0.0 ^b^	*p* = 0.987
	female	11	35.48	12	35.29		
Socioeconomic status	below average	3	9.68	0	-	χ^2^_(2)_ = 3.50 ^b^	*p* = 0.174
	average	14	45.16	16	47.06		
	above average	14	45.16	18	52.94		

Attention deficit hyperactivity disorder (ADHD); a—Independent-samples *t*-test; b—Chi-squared test for independence.

**Table 2:** Comparison of participation patterns (frequency and involvement), desire for change, and the overall environmental support in the different settings between the two groups.

Measure	Setting	With ADHD (*n* = 31)	Without ADHD (*n* = 34)	F_(1, 63)_	η^2^
		mean (SD)	mean (SD)		
Frequency (8-point scale)	Home	5.77 (0.66)	6.08 (0.45)	5.10 *	0.08
	School	4.60 (1.28)	4.35 (0.93)	0.85	0.01
	Community	3.17 (1.06)	3.61 (0.80)	3.60	0.05
Involvement (5-point scale)	Home	3.50 (0.60)	4.30 (0.39)	41.55 ***	0.40
	School	3.89 (0.86)	4.35 (0.59)	6.40 **	0.09
	Community	3.74 (0.91)	4.30 (0.54)	9.12 **	0.13
		mean in percent (SD)	mean in percent (SD)		
Desire for change	Home	68.92 (24.21)	47.35 (23.23)	13.43 ***	0.18
	School	47.58 (33.59)	32.45 (30.45)	3.63	0.05
	Community	42.90 (24.56)	27.64 (22.47)	6.85 **	0.10
Overall environmental support	Home	83.78 (8.58)	94.11 (6.38)	27.52 ***	0.31
	School	82.54 (10.67)	94.89 (6.76)	30.98 ***	0.33
	Community	96.42 (12.66)	94.82 (7.67)	10.45 **	0.14

Notes: * *p* < 0.05, ** *p* < 0.01, *** *p* < 0.001.

**Table 3:** Pearson’s correlations between environmental support and participation patterns (frequency and involvement) among children with and without attention deficit hyperactivity disorder (ADHD).

	Overall Environmental Support	
	Children with ADHD (*n* = 31)	Children without ADHD (*n* = 34)
Frequency		
Home	0.06	0.23
School	0.27	0.3
Community	0.03	0.24
Involvement		
Home	0.38 *	0.37 *
School	0.49 **	0.2
Community	0.47 **	−0.02

Notes: * *p* < 0.05, ** *p* < 0.01; Pearson’s correlations (r).

## Data Availability

The dataset generated and analyzed during the current study is available to the first and last authors, but are not publicly available due to ethical guidelines.
